# Examining the Impact of Visual Perturbation Caused by Virtual Reality on Postural Stability

**DOI:** 10.5114/jhk/197097

**Published:** 2025-07-21

**Authors:** Sayedmohsen Mortazavi Najafabadi, Mohammed Najafi Ashtiani, Bartłomiej Zagrodny, Dariusz Grzelczyk

**Affiliations:** 1 Department of Automation, Biomechanics and Mechatronics, Lodz University of Technology, Lodz, Poland.; 2 Department of Physical Therapy, Faculty of Medical Sciences, Tarbiat Modares University, Tehran, Iran.

**Keywords:** static balance, postural balance, visual disturbances, optic flow

## Abstract

Application of virtual reality has gained traction as a promoting tool in rehabilitation techniques, particularly in the enhancement of postural balance. This study aimed to explore the effects of various virtual reality environments on the postural control of young adults. Twenty-five active collegiate students (14 females and 11 males) participated in this cross-sectional investigation. Participants experienced four distinct virtual environments: (1) a wall approaching at a constant speed, (2) a wall accelerating towards them, (3) a tilting wall, and (4) a wall that returns after tilting. Postural sway was assessed using a pedobarographic platform, and the center of pressure matrices was computed. A one-way repeated measures analysis of variance was conducted to analyze the postural sway metrics across the reference and the four experimental conditions. Significant alterations were detected in maximum sway excursion (p = 0.004), and sway variability (p < 0.001) in the anterior-posterior axis, as well as sway variability in the medial-lateral axis (p = 0.022). Pairwise comparisons indicated that the environments featuring the accelerating wall and the returning wall upon tilting produced the most significant changes. Conversely, maximum sway velocity and sway area variables did not exhibit significant changes in either direction. The findings suggest that virtual reality environments, particularly those simulating tilting or movement, considerably challenge postural control without inducing simulator sickness. Furthermore, virtual reality offers a cost-effective means to simulate realistic visual disturbances, which can be beneficial for designing balance training based on virtual reality like exergame therapies.

## Introduction

Postural control refers to the essential skills needed to sustain balance and coordinate movements of the body in various positions, which are crucial for performing both everyday activities and more complex tasks (Anne Shumway-Cook, 1995). The concept of postural stability is intricate and can be characterized as the ability to return to a primary or secondary balanced position following a disturbance (Wagner and Blickhan, 1999; Wang et al., 2024; Wilczyński et al., 2022; Wodarski, 2023). Research has further defined postural stability as the ability to control one's center of mass (CoM) within the boundaries of the base of support, thus averting falls (Maki and McIlroy, 1996). Clinically, it can be succinctly articulated as the ability to remain upright without falling or development of pain (Panjabi, 1992). The maintenance of postural control relies on the integration of sensory information from the vestibular, proprioceptive/somatosensory, and visual systems within a closed feedback loop, with the visual system being particularly significant in the process of maintaining balance (Lin et al., 2021), and playing a key role in the postural balance process (Taneda et al., 2021). Any dysfunction or erroneous input from these sensory systems can compromise postural control, leading to balance disturbances, falls, and potential injuries. Effective postural management requires timely and appropriate muscle responses to adapt to changes in the environment (Allison et al., 2006; Bhende et al., 2024; Woollacott, 2000).

The extent to which sensory systems contribute to stability varies depending on the situation. However, in the 1970s Lee and Aronson showed that vision had a major impact on postural control. Their tests employed a mobile chamber to demonstrate the influence of visual information on postural regulation. In instances where there was a discrepancy between visual input and the body's spatial sense of position, the body tended to prioritize information derived from proprioception and vestibular senses (Lee and Aronson, 1974). Dijkstra et al. (1994) used a video projector and showed that postural sway had a consistent temporal correlation with moving visual surroundings, which was affected by the distance of visual stimuli (Dijkstra et al., 1994). Hoshikawa's research (1999) delved deeper into the environmental impact by investigating the consequences of tilting rooms. Participants positioned themselves within a revolving chamber, resulting in heightened bodily movement and emphasizing the significance of the visual system in maintaining equilibrium. This rotation caused a shift in the CoM towards the edge of the base of support, which then activated compensatory postural adjustments through reflexes in the muscles of the lower extremities (Hoshikawa, 1999). Optical flow, which represents the movement of the surroundings in relation to a person who is not moving, also influences postural stability. It is essential for determining one's position and direction inside a given space (Phillips et al., 2022). An experimental configuration using force plates to quantify the center of pressure (CoP) revealed that older individuals were more vulnerable to variations in the visual field (Wade et al., 1995). The study conducted by Sundermier et al. (1996) highlighted that older persons experiencing balance issues were more vulnerable to optical flow.

As mentioned earlier, the moving room configuration is useful for experiments, but it requires a lot of area and construction. Computer-controlled motors can offer exquisite control to perturbations (Godoi and Barela, 2016), but they also increase cost and space. It can limit visual disruptions and movement duration, and it can block subjects' lines of sight. Although optical flow affects postural control, other components of optical flow and vision in postural stability have received less attention including the following: direction (anterior/posterior, mediolateral, tilting), distance, the optical flow pattern, perturbation velocities, and ambient textures and signals (Godoi and Barela, 2016; Raffi et al., 2017). Freeman et al. (2023) examined how perturbing the moving wall paradigm caused the wall to move toward and away from the person with constant velocity. The experiment showed that this caused postural difficulties, especially when the moving room moved unexpectedly and toward the occupant. In addition to studies focused on healthy individuals, research has also explored the effects of optical flow on patients with cerebral palsy who exhibited greater dependence on visual cues for maintaining balance while walking, demonstrating increased sway and diminished ankle response in comparison to their typically developing peers (Sansare et al., 2022). Experiments conducted by Michnik et al. (2016) and Jurković et al. (2017) have demonstrated that visual disruptions, such as changes in frequency and environmental conditions, have a substantial impact on stabilographic parameters. This emphasizes the crucial importance of visual inputs in preserving postural balance (Jurkojć et al., 2017; Michnik et al., 2016).

Recent advancements in visual perturbation research involve the use of virtual reality (VR) environments with head-mounted displays (HMDs) (Moon et al., 2021; Rosiak et al., 2024). HMDs provide the advantage of being portable and flexible, enabling users to modify virtual worlds to accommodate different disruptions. Although HMDs offer benefits compared to conventional moving rooms, their effect on controlling the CoP during optical translational movement is not well comprehended (Beani et al., 2022). Besides, previous studies have investigated the control of the CoP majorly during the optical translational movements. However, sensory feedback sources of postural control rely to a greater extent on such types of movements as rotational and reverse motions. The literature has rarely focused on VR-based visual movements, their moving details, and their comparison. Therefore, the purpose of this study was to develop and test a complete VR environment simulating the moving room with constant and accelerating velocity, as well as a tilting room, to examine postural control in perturbed environment. Additionally, we aimed to determine which of the two distinct disturbance types would influence a person's postural control most. It was hypothesized that sudden visual perturbations presented in a virtual reality environment would affect balance metrics during quiet standing.

## Methods

### Participants

Twenty-five students of the Lodz University of Technology volunteered for this study. Table 1 presents their basic anthropometric data. G*Power statistical software (v3.1) and analogous prior research (Freeman et al., 2023) set the study's participant effect size at 0.4. The exam was completed by 25 people. The inclusion criterion was age between 18 and 40. On the other hand, the exclusion criteria were smoking and/or alcohol use before examination, recent injuries and/or surgeries, and neurological, metabolic, and musculoskeletal disorders as well as any joint replacement. Additionally, each participant completed a simulation sickness questionnaire (SSQ) regarding their virtual environment (VE) exposure, with a score > 5 (see study protocol section) considered an exclusion criterion. The study was approved by the Lodz University of Technology Research Ethics Committee (protocol code: 2/2023; approval date: 30 March 2023) and was conducted in the laboratory of biomechanics at the Lodz University of Technology. Participants provided written and verbal consent after receiving detailed information about the aim, scope, and procedures of the experiment.

**Table 1:** Participants’ basic anthropometric data (mean ± SD).

	Sex	Age (years)	Body mass (kg)	Body height (cm)	BMI (kg/m^2^)
Participants (N = 25)	Male = 11 Female = 14	22.4±4.5	70.2±15.6	171.1±7.1	23.8±3.7

### Equipment

A pedobarographic force plate (Footscan® system, RSscan International, 12288 sensors in a 192 × 64 matrix, scanning 200 Hz) was used to measure CoP excursion. Virtual environments (VEs) were created using Unity 3D (Unity Technologies, v2021.3, San Francisco, CA). Afterwards, a head-mounted display (Oculus Rift-S, v6, Facebook Technologies, CA, 94025 USA) was used to immerse participants in the virtual reality experience.

### Study Protocol

**Figure 1:**
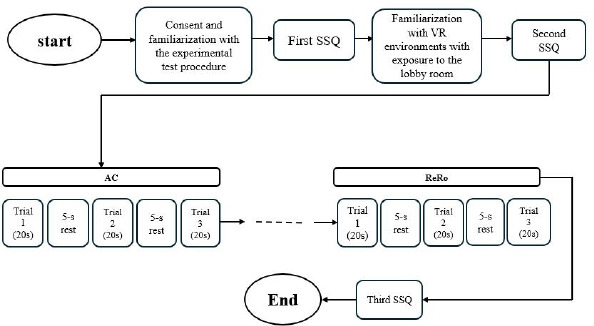
Flow chart of the study experimental procedure.

The study protocol was thoroughly explained to the participants upon their arrival at the laboratory. A simulation sickness questionnaire (SSQ) (Kim et al., 2018) that comprises a total of sixteen questions centered on simulator sickness, to which response scores range from 0 (none) to 3 (severe), was the only subjective questionnaire used in this investigation. The SSQ was used to determine further eligibility for the study, and participants with an SSQ score greater than five and any associated risk factors were excluded. The experimental protocol is presented in Figure 1.

**Figure 2:**
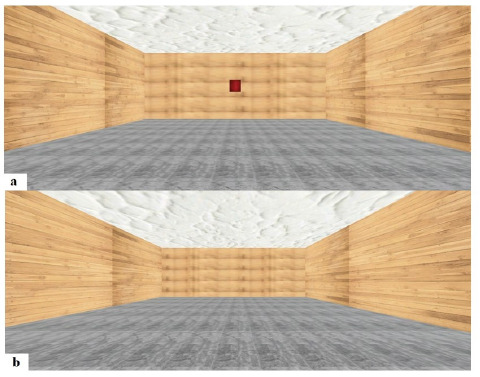
Virtual Environments: (a) lobby and transition area with a red box that, when viewed, moved into the testing environment; (b) closed room testing environment.

The pedobarographic and VR systems, using software instructions and hand-held sensors, respectively, were calibrated before testing. Participants were instructed to stand with their eyes open during quiet standing with no VR (NV) for 10 s barefoot on the force plate after being briefed about the overall procedure of the experimental test following a five-minute rest post-familiarization. The lobby area virtual environment (VE), serving as familiarization, was the first VE encountered by participants (Figure 2a). This familiarization, lasting 10 s only, was done to acquaint participants with the VE and allow them to practice gaze-based navigation skills. A red square appeared in the center of the lobby area, and participants were instructed to stare at it for ten seconds until it disappeared. Participants were then exposed to a new testing VE without the red square (Figure 2b). They were instructed to stand as upright as possible, without swaying, and to look straight at the front wall of the room. No additional information was provided to the participants except for these instructions. Each VR task was accompanied by verbal instructions.

**Figure 3:**
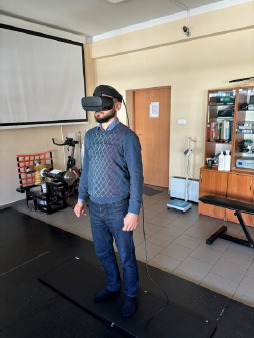
One of the participants in the experimental Vroom paradigm setup is standing on the Footscan system balance platform while donning a VR headset.

The study participants were exposed to four distinct environments: (1) a moving wall that moved at a constant speed of 9 m/s towards the participant (MW); (2) a moving wall that could be accelerated to alter the movement's speed (AC), with initial acceleration of 1.8 m/s^2^ for the first 0.5 s and second acceleration of 8 m/s^2^ for 1 s. In both scenarios, the wall distance travelled remained constant (1 m to the participant); (3) a tilted room that rotated at a speed of 20°/s with a target angle of 45° (Ro); and (4) an environment that rotated in the same manner as before, but the wall returned to its starting position once it reached the target degree (ReRo) (Figure 3).

The room was fully illuminated, with no shadows present. The participant's field of view was 110°, and the refresh rate was 90 Hz. To introduce an element of surprise in the tests, participants were given a 10-s preview of a red cube in the lobby environment before entering the test environment. Upon entry, participants encountered the perturbation environment after two, three or five seconds (chosen randomly by the researcher), constituting three trials. Each participant underwent a total of 15 tests across five distinct settings in this manner. A 5-s rest interval was provided between each trial.

### Data and Statistical Analysis

Although posturographic data were acquired meanwhile the test, before and after the application of perturbations, analyses were performed for time intervals exactly after the perturbation to seek pure transient response of the body against the visual disturbances. Matlab (MathWorks Inc., MA, USA) was utilized to calculate postural balance metrics, including maximum sway velocity (cm/s), maximum sway excursions (cm), sway variability (cm), and the total sway area (cm^2^). The definitions of these variables are elaborated upon in the paper of Babayi et al. (2023). In brief, maximum sway velocity was the maximum value of the instantaneous time derivative of the CoP displacement in both AP and ML directions. Maximum sway excursion was the greatest absolute CoP displacement in AP and ML directions. Also, this study assumed standard deviation of the CoP sway in AP and ML direction as the sway variability parameter (Reisi et al., 2024). The sway area was defined as the area of an ellipse that encompassed at least 95% of the sway data points.

One-way repeated measures analysis of variance (ANOVA) was employed to examine the relevant postural sway variables between the reference (NV) and the four experimental conditions (MW, AC, ReRo, and Ro). Post-hoc pairwise comparisons were conducted using the Bonferroni correction factor to further explore any significant main effects. Statistical analyses were performed using IBM SPSS (version 20), with the significance levels of all analyses set at α of 0.05. The effect size of the difference was determined by computing eta-squared (η^2^), which was interpreted as: η^2^ > 0.01 (small), > 0.06 (medium), or > 0.14 (large) (Cohen, 1988).

## Results

No participant was excluded from the analyses based on their exposure to the virtual environment, as determined by the results obtained from the SSQ.

**Figure 4:**
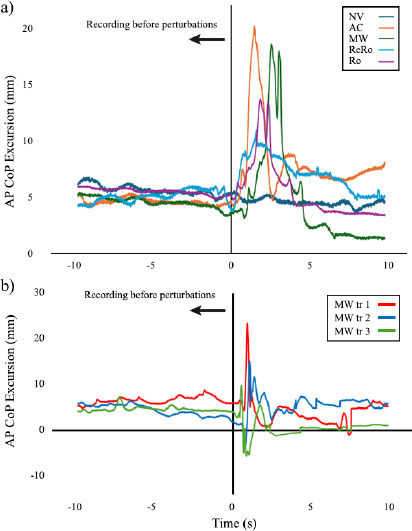
Sample graphs of a) time-dependent variations of anterior-posterior (AP) CoP variations for five different visual environments including an accelerated moving wall (AC), a constant-velocity moving wall (MW), a returning rotation (ReRo), rotations (Ro), and no virtual reality change (NV) for subject #9, and b) three consecutive trials of MW for subject #2. Time at zero denotes the moment of perturbation application, because the perturbation was unexpected; it did not occur at a specific time, but for consistency, it was plotted in a synchronized manner.

**Figure 5:**
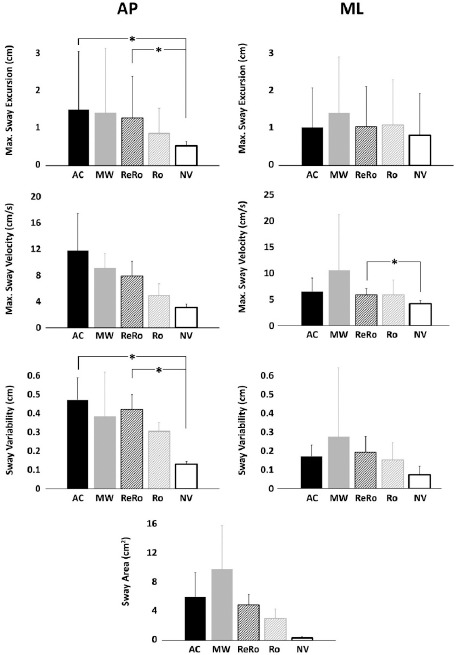
CoP-related metrics of balance for five different visual environments including an accelerated moving wall (AC), a constant-velocity moving wall (MW), a returning rotation (ReRo), rotations (Ro), and no virtual reality change (NV) in a) anterior-posterior (AP) and b) medial-lateral (ML) directions with means and error bars as standard deviations. The asterisks * denote significant differences (*p* < 0.05).

Figure 4 plots time-dependent variations of AP CoP variations for five different visual environments. In the anterior-posterior direction, virtual environments significantly influenced CoP-related measures of balance of maximum sway excursion (F(4,21) = 6.2, *p* = 0.004, η^2^ = 0.21) and sway variability (F(4,21) = 7.4, *p* < 0.001, η^2^ = 0.24). Pairwise comparisons of the significant main effects exhibited significant increases in maximum sway excursion in AC (*p* = 0.041) and ReRo (*p* = 0.021) virtual environments compared to the no virtual condition (NV). Also, sway variability of the CoP was significantly increased in AC (*p* = 0.020) and ReRo (*p* = 0.010) virtual environments compared to the NV condition. However, the sway area and maximum sway velocity variables did not significantly change (Figure 5 AP).

In the medio-lateral direction, our results indicated a significant increase in the main effect only for sway variability among the testing environments (F(4,21) = 3.87, *p* = 0.022, η^2^ = 0.14), but not for maximum sway excursions. Despite the fact that a significant change in the sway variability was seen in virtual reality environments, in our statistical pairwise comparison, no significant difference was seen among different environments. Also, the pairwise comparisons of the significant main effect revealed that the ReRo environment induced greater maximum sway velocity than the NV environment with corresponding *p* values of *p* = 0.029 (Figure 5 ML). In this direction, as in the anterior-posterior direction, the sway area and maximum sway velocity did not change significantly.

## Discussion

The objective of this work was to examine the effects of temporary virtual reality perturbation on the characteristics of postural sway. The metrics included sway variability, maximum sway excursion, and the sway area. These variables were important indicators of how the body responded to the visual perturbation while keeping balance. Our findings confirmed the influence of application of visual perturbation via virtual reality environments on the control of posture. It was also demonstrated that different virtual environments required distinct strategies for maintaining balance. Sensory systems and cerebral pathways interact complexly to perceive acceleration, constant velocity, and rotation. The visual cortex processes constant velocity and acceleration (Maunsell and Van Essen, 1983). The semicircular canals and otolith organs of the vestibular system detect linear acceleration and rotation (Angelaki and Cullen, 2008). Exposure to VR with optic flow stimuli can reduce vestibular sensitivity, particularly when visual and vestibular cues are congruent. This indicates that the brain may down-weight vestibular signals to resolve sensory conflicts (Gallagher et al., 2020). Individuals with Parkinson's and unilateral peripheral vestibular dysfunction show increased visual dependency for balance when exposed to VR-based visual perturbations. This dependency is linked to impaired vestibular function and proprioception (Hawkins et al., 2021).

Significant changes in postural balance metrics were observed in the current investigation between the quiet standing condition and unexpected VR situations. Specifically, when compared to NV, sway variability and maximum sway excursions were considerably higher in the AC and ReRo environments. This result implies that a person's instability is more affected by rapid movement and the return movement of a rotating room than in other environments. This can be explained by the increased challenge posed to a person's control system in maintaining stability under such conditions. No variations were observed in maximum sway excursions and medial-lateral sway variability among the expected circumstances, which is in contrast to the anterior-posterior findings.

Our results align with those of prior studies (Chander et al., 2019; Freeman et al., 2023), which investigated unexpected moving room perturbations. The perturbations in the current study were applied along the anterior-posterior axis, which may have caused the participants to shift their CoM correction in the anterior direction. There were no significant variations in displacement between reference and unexpected visual disturbance situations because the disruption only affected the anterior-posterior axis, maintaining medial-lateral stability throughout the postural disruption. Visual perturbations in VR can significantly increase joint excursions, including the ankle, the knee, the hip, the spine, and the shoulder, especially when using a first-person perspective compared to a third-person perspective (van der Veen et al., 2020). Higher velocities of visual scene movement in VR lead to increased angular displacements in the hip, knee, and ankle joints, indicating that visual cues drive postural adjustments (Dokka et al., 2009). Visual interference, such as the elimination of vision, increases ankle rotations due to the lack of visual feedback, while cognitive loads enhance knee and hip powers, but do not significantly affect the ankle (Ashtiani and Azghani, 2018).

In the current study, no significant change was found in the sway area among VR environments compared to the NV condition in both the anterior-posterior and medial-lateral directions. The absence of significant differences in postural sway values between NV and VR environments contradicted our original hypothesis, which suggested negative impacts on postural stability or increased postural sway when exposed to VEs and the HMD. Prior research has reported such decrements in postural stability in VR-generated VEs compared to real environments due to distorted visual feedback from the VE and changes in the visual field due to immersive VR (Assländer et al., 2015; Chander et al., 2021). Wodarski et al.'s (2021) research corroborated our results, indicating that conventional stabilometric assessments might be insufficient for identifying the nuanced impacts of destabilizing stimuli on postural stability, particularly in the presence of conflicting sensory inputs (Wodarski et al., 2021).

Exploring internal and external attentional focus and VR exposure helps explain the current study's NV condition findings. The VE was more organized and provided fewer distractions compared to the lab setting, even though all tasks required participants to stand straight and look at the wall. In VR, participants' external focus on the controlled VE's center point can improve postural stability. In contrast, the non-VR setting's real-world openness may lead to a less precise focus on the front wall's center. The absence of visual virtual disturbances may have increased postural sway in the VR environment (Wulf et al., 2003). Furthermore, Imaizumi et al. (2020) postulated that body sway was influenced by the weight of the head mounted display and sensory input. Due to familiarity and the 10-s start time of the perturbation, participants probably overcame this influence over time, diminishing its impact. Our study did not find such significant changes, in contrast to earlier research (Freeman et al., 2023; Imaizumi et al., 2020) which shows that virtual reality environments significantly increase maximum sway velocity.

The study conducted by Michnik et al. (2016) examined the effects of environmental translation and rotation (with amplitudes of 0.3 m and 0.05 m, respectively) on two distinct scenery types (open and closed) subjected to two categories of disturbances: an increase in frequency from 0.7 Hz to 1.4 Hz and a decrease in frequency from 1.4 Hz to 0.7 Hz. Those authors showed that frequency perturbations had a significant effect on stabilometric variables, particularly at low frequencies. This finding is supported by another experiment conducted by Wodarski et al. (2023). While we did not investigate the impact of frequency perturbations in our study, our findings are not in conflict with theirs, as we employed a different kind of perturbation (Michnik et al., 2016; Wodarski et al., 2023). Another significant finding reached by the authors of the aforementioned study was that participants gradually developed tolerance to visual disturbances. In order to tackle this problem, we opted to administer our tests randomly. According to Wodarski et al. (2023), the CoP displacement drifts gradually. Thus, diagnostic stabilographic tests should last longer than 30 s. Conversely, our study examined how a single, short disturbance—a fast, unexpected movement of the surroundings—would affect postural stability. The literature has shown that slower optical flow velocities in virtual reality environments significantly impact user's postural balance, potentially increasing the risk of falling (Santoso and Phillips, 2020). Weaker responses may arise from biomechanical constraints that restrict the human body from swaying at higher velocities (Morasso and Sanguineti, 2002). Additionally, faster visual motion inherently results in increased velocities of the visual scene or optic flow on the retina. When these velocities surpass a certain threshold, the perception of self-motion diminishes, as the visual system ceases to associate the movement with self-motion (Day et al., 2016). A similar saturation effect is observed when range of movement is augmented while velocities remain unchanged, which also leads to an increase in optic flow velocity (Morasso and Sanguineti, 2002). This requires short-term data analysis. One important discovery emphasizes the need for virtual reality therapy safety protocols. Our experience suggests using personal protection, harnesses, soft pads or other safeguards when testing any movement that could cause the individual to lose balance (Wodarski et al., 2020).

## Study Limitations and Future Work

This study faced some limitations. First, the protocol was designed to acquire data in three trials to minimize uncertainties by averaging between the outcomes. This might either reduce the unexpectedness of the perturbations or cause adaptations, although start times of trials were unpredictable to the study participants and the order of environments was fully randomized. Second, use of the HMD might have affected the participants’ response since previous research has shown that HMDs limit lateral movement and flatscreen virtual reality increasing anterior-posterior movement (Lott et al., 2003). Finally, this study included healthy young people only, thus future investigations involving subjects with balance disorders (such as neurological pathologies) and/or the elderly are recommended. Besides, kinematic measurements of body sway, specifically to calculate CoM excursions, would confer more comprehensive postural control indices.

## Conclusions

In this study, virtual reality was used to simulate moving rooms, and tilting room tests were used to examine postural control behavior depending on postural data. We found that virtual reality settings affected postural balance, especially in scenarios with fast room movement and return rotation. Due to the physical weight and focus that people maintain in virtual reality, the sway area variable may not be a suitable evaluation measure in this experiment. Virtual settings can provide a more realistic experience without simulator sickness, boosting balance assessment confidence.
